# Non-covalent SUMO interactions with ligases and effectors: SUMO-interacting motifs and beyond

**DOI:** 10.1042/EBC20253039

**Published:** 2025-11-17

**Authors:** Aanchal Mishra, El Hadji Cisse, Marcin J. Suskiewicz

**Affiliations:** 1 Centre de Biophysique Moléculaire (CBM), Orléans, CNRS, UPR 4301, France; 2 École Doctorale “Santé, Science Biologique & Chimie du Vivant” (ED549), Université d'Orléans, France

**Keywords:** post-translational modifications (PTMs), small ubiquitin-like modifier (SUMO), SUMO-interacting motifs (SIMs), SUMOylation, WD40 repeat domains, ZZ domains

## Abstract

SUMOylation, a protein post-translational modification (PTM) involving the covalent attachment of small ubiquitin-like modifier (SUMO), regulates a wide range of cellular processes. The key hallmark of SUMO that distinguishes it from ubiquitin is the hydrophobic groove that binds short linear motifs known as SUMO-interacting motifs (SIMs), which are found across a broad spectrum of partners, including SUMO E3 ligases and downstream effector proteins such as transcription factors, DNA-repair proteins, ubiquitin E3 ligases and cell-signalling components. In addition, various effectors interacting in a SIM-independent manner have been reported. In this review, we summarise the current understanding of non-covalent SUMO interactions mediated by SIMs and other, alternative SUMO-binding elements. Focusing on the evolution and structural basis of these interactions, we discuss the methodological approaches used in the field, outline emerging mechanisms and concepts and highlight key open questions.

## Introduction

SUMOylation, an essential eukaryotic post-translational modification (PTM) related to ubiquitylation, proceeds through an enzymatic cascade composed of a single E1 enzyme, a single E2 enzyme and a limited set of E3 ligases [1,2]. SUMOylation can be removed by SUMO-specific proteases, which makes it a reversible and highly dynamic process [3].

The function of SUMO (small ubiquitin-like modifier) relies not only on its ability to be covalently ligated, but also on its diverse non-covalent interactions. These include, first, the interactions with core enzymes catalysing the conjugation and deconjugation processes, namely E1, E2 and SUMO-specific proteases. Second, SUMO can also interact with SUMO E3 ligases, auxiliary proteins that act as scaffolds to accelerate, and refine the specificity of, the discharge of SUMO from the E2 enzyme onto a substrate. Third, the produced SUMO modifications (including polySUMO chains and mixed SUMO–ubiquitin chains) can mediate interactions with various downstream effectors, such as transcription factors, chromatin remodellers, DNA-repair proteins, ubiquitin E3 ligases or cell-signalling components. This review discusses the interaction with SUMO E3 ligases and downstream effectors, complementing the accompanying review [4], which covers the core SUMO:enzyme interactions.

While SUMO interactions with enzymes tend to rely on extended interaction surfaces, most remaining interactions are mediated by specific short linear motifs known as SUMO-interacting motifs (SIMs) [5–7]. These motifs, typically found in protein regions that are disordered in the unbound state, bind a specific groove on SUMO *via* β-strand addition in either a parallel or an anti-parallel manner and are present in a wide range of proteins, including SUMO E3 ligases and the various downstream effectors mentioned in this review. In addition to SIM-dependent binding, some effectors might bind SUMO in SIM-independent manners. The possibility of such alternative SUMO-binding modes being considerably more common than is currently appreciated is supported by recent proteomics studies designed to identify SIM-independent interactions [8,9].

In this review, we discuss the binding elements mediating non-covalent interactions with SUMO beyond the specific surfaces of core SUMOylation enzymes. A central place will be devoted to SUMO:SIM interactions, but emerging SIM-independent interaction types will also be discussed. We examine the structural basis, evolutionary origins, regulation and functions of these binding events, alongside experimental and computational methods used to identify and characterise them. Unless otherwise specified, SUMO residue positions refer to human SUMO1 numbering. We use ‘–’ for stable covalent bonds and ‘∼’ for labile thioester bonds, while non-covalent complexes are indicated with ‘:’.

## SUMO properties and the variety of SUMOylation signals

An extensive discussion of the evolution, structure, surface properties, and the (de)conjugation cycle of SUMO can be found in the accompanying review [4]. Briefly, SUMO is a member of the broader ubiquitin-like (UBL) family and retains the β-grasp fold common to other UBLs and certain prokaryotic sulphur-carrier proteins [10–12]. It exists as a single orthologue in yeast but has diversified into several paralogues (SUMO1 to SUMO5) in humans, of which SUMO1, SUMO2 and SUMO3 are the most widely expressed and conjugated to substrates [13]. SUMO2 and SUMO3 are nearly identical to each other but considerably distinct from SUMO1. Beyond the central β-grasp fold, SUMO features flexible N- and C-terminal tails, of which the first is particularly long, encompassing around fifteen amino-acid residues [14]. SUMO features a specialised SIM-binding groove, which is its primary site for non-covalent interactions. SUMO is covalently attached to Lys residues on substrates through an isopeptide bond, in a process that minimally requires a SUMO-specific E1 enzyme (the SAE1:SAE2 heterodimer) and a SUMO-specific E2 enzyme (UBC9), and can be accelerated by a SUMO E3 ligase [2].

### A ‘SUMO code’?

One aspect of the function of SUMO that has not been much discussed in the accompanying review [4] and which is of particular interest to the discussion of SUMO:effector interactions is the possibility of multiple SUMO molecules being simultaneously conjugated to the same substrate in various configurations. Like ubiquitin, SUMO can be attached as a single unit to one site (monoSUMOylation) or as multiple units – and, in the latter case, either individually to multiple sites (multiSUMOylation) or as chains of SUMO molecules to one or multiple sites (polySUMOylation). PolySUMOylation involves the attachment of succeeding SUMO molecules to Lys residues on preceding SUMO molecules. The dominant chain type in humans appears to be that composed of SUMO2/3 units connected *via* Lys11 [15,16] but other chain types – involving various Lys residues within SUMO2/3 (mainly Lys5, 7, 32/33, 41/42 and 44/45) and/or SUMO1 (predominantly Lys7) – have also been detected [16,17]. Some results suggest that SUMO1 might often feature as the last, capping unit on SUMO chains that are otherwise mostly composed of SUMO2/3 [16].

Beyond SUMO chains, a particularly intriguing form of modification involves mixed SUMO–ubiquitin chains, where ubiquitin is conjugated to SUMO chains, or vice versa [18]. One known source of such mixed chains is the action of SUMO-targeted ubiquitin E3 ligases (STUbLs), a class of ubiquitylating enzymes that target polySUMOylated substrates to trigger their proteasomal degradation [19–26]. Other UBL proteins might also form mixed chains with SUMO, for example NEDD8, which has been reported to form NEDD8–SUMO2 hybrids [27]. Further complexity of SUMO signals might be introduced by the presence of other PTMs on SUMO, notably phosphorylation [16,28] and acetylation [29].

It is currently unclear to what extent different types of SUMOylation – mono- vs polySUMOylation, various polySUMO chains with different compositions (including different SUMO paralogues and ubiquitin or other UBL proteins), linkage types (the specific Lys residues involved) and topologies (linear vs branched), presence of small PTMs on SUMO, etc. – give rise to a functional ‘SUMO code’ analogous to the ‘ubiquitin code’ postulated in the ubiquitin field [30,31]. If these diverse SUMO signals do exhibit some degree of code-like functional specificity, one would expect the existence of mechanisms favouring specific encoding of functional cues and specific differential decoding – the first process involving the E2 enzyme and E3 ligases, the latter – readers capable of recognising a particular SUMO signal. So far, there are indications that some SUMO readers and erasers – notably the mentioned STUbLs or certain SENP proteases – evolved to preferentially recognise polySUMO as opposed to monoSUMO by virtue of having multiple SIMs [22,32–37]. Some proteins of this type might favour either homogeneous SUMO2/3 chains or SUMO1-capped SUMO2/3 chains [36]. Moreover, high-throughput analyses of SUMO binders using both monoSUMO and head-to-tail SUMO fusions that mimic natural polySUMO chains have revealed that some binders preferentially interact with the latter [38,39]. Also, a modular ubiquitin–SUMO reader, RAP80, appears to preferentially bind to a particular SUMO–ubiquitin chain topology [40]. We will briefly return to this topic, which is poorly understood so far, later in the text.

## Techniques for studying non-covalent SUMO interactions

Possible approaches for identifying and characterising non-covalent SUMO interactions range from high-throughput methods for detecting potential binders, through techniques enabling cellular and *in-vitro* validation, to quantitative methodologies allowing measurement of interaction parameters such as equilibrium dissociation constant (*Kd*) and kinetic association and dissociation constants. These efforts culminate in high-resolution structural analyses that visualise these interactions, as well as biological experiments that assess their physiological relevance. Bioinformatic tools, including recent structure prediction methods, allow bypassing some of these steps by suggesting possible interaction partners and producing structural models of the complexes formed.

### Identifying potential SUMO binders

Especially in earlier periods, yeast two-hybrid (Y2H) screening – a classical tool for probing protein:protein interactions – has been extensively used as a medium- or high-throughput technique for identifying potential SUMO-binding proteins [41,42]. Another, increasingly more popular, first-pass approach has been pulldown or affinity purification coupled with mass spectrometry. SUMO is often tethered to beads using a His₁₀ tag, with elution of non-covalent interactors performed using urea [38], although other approaches are possible as well [43]. These strategies can be used to identify binders that preferentially interact with specific SUMO paralogues or with SUMO chains, in the latter case by using baits that mimic SUMO chain fragments. Very recently, using SUMO2 mutated at the SIM-binding groove, Vertegaal and colleagues were able to focus this type of analysis on potential SIM-independent interactions [9]. A further recent technique for the high-throughput identification of SUMO binders involves a protein microarray blotted with specific SUMO paralogues or polySUMO2 chains [39,44]. In these approaches, bound SUMO is either fluorescently labelled or detected with antibodies.

### Capturing transient interactions

While pulldown coupled with mass spectrometry remains a cornerstone method for identifying potential SUMO-binding partners from cell lysates, transient or low-affinity interactors might require different approaches. One such complementary approach is proximity-dependent biotinylation, which allows mapping of the protein environment – including transient binding partners – in a cellular context. A sophisticated version of this approach, called SUMO-ID, has been developed by Barrio, Sutherland and colleagues [45]. Here, two inactive fragments of the TurboID biotinylation enzyme are fused to SUMO and a protein of interest, respectively, such that a functional TurboID is reconstituted when the protein of interest becomes SUMOylated or interacts with fused SUMO. This results in the biotinylation of proximal proteins, potentially including specific non-covalent interactors of the SUMOylated form of the protein of interest.

Another strategy for stabilising transient interactions involves genetic code expansion to introduce a cross-linking warhead [46]. This approach has an added advantage of preferential targeting of proteins that interact with a specific part of SUMO, depending on the cross-linker location. Recently, by incorporating a photoactivatable cross-linking residue either near the SIM groove or on the opposite face of SUMO1, Mootz and colleagues identified large numbers of potential binders with preference for a given region of SUMO [8].

### Validation and characterisation using cellular and *in vitro* techniques

Validation and further investigation of potential interactions identified by the approaches described above are ideally pursued using both cellular and *in vitro* techniques. In cells, microscopy-based methods [43,47–49] and pulldown/co-immunoprecipitation followed by immunoblotting [43,47,50,51] along with functional readouts related to a specific process to which a given interaction contributes – help confirm specificity and physiological relevance. In pulldown-based approaches, a SIM peptide can be added as a competitor to demonstrate that the given binding event depends on the SIM-binding groove [43]. While some insights can be gained from studies involving transient overexpression and/or fluorescent tagging, working with endogenous, untagged proteins is ideal.

Given the essentiality of SUMOylation in yeast and the relative simplicity of yeast as a model organism, the impact of mutations in some SUMOylation machinery components can be tested by monitoring yeast growth and viability [52,53]. Interestingly, a mutational screen performed in *S. cerevisiae* has confirmed the collective importance of SUMO:SIM interactions, with mutations of several residues within the SIM-binding groove producing lethal phenotypes [54].

*In vitro*, recombinant proteins or fragments (including synthetic peptides) can be used to validate interactions – initially with semi-quantitative methods such as *in vitro* pulldown assays [35,43,50,55,56], followed by quantification of thermodynamic and kinetic parameters, particularly *Kd* – using techniques like isothermal titration calorimetry (ITC) [33,57–61], surface plasmon resonance (SPR) [39,43,62], bio-layer interferometry (BLI) [39,63], nuclear magnetic resonance (NMR) spectroscopy [64,65], and others. While these assays are often combined with mutagenesis of selected residues, a more systematic exploration of each residue within a peptide can be performed in a semi-quantitative manner using a peptide microarray [64].

### Structural biology

In addition to validating an interaction, mapping it to specific regions on SUMO and its partner is a key part of the analysis. Mapping can be performed by monitoring chemical shift perturbations using NMR or by performing a series of binding experiments with various fragments/mutants. A recent alternative approach involves carbene footprinting, in which SUMO-interacting regions are identified as those protected from reaction with a diazirine compound in the presence of SUMO [39]. Ultimately, high-resolution structural methods – such as NMR [35,64] X-ray crystallography [61,66] or, for larger complexes, cryo-electron microscopy (cryo-EM) [67,68] – can provide conclusive and detailed insights into the interaction interface.

### Computational tools

In addition to the experimental approaches described above, an important role in the field has been played by computational methods.

Bioinformatic approaches have been used to generalise from known, experimentally validated SIM sequences to predict new SIMs in proteins of interest. The three main currently available tools – each of them available as a web server, and the second additionally as a software with documentation for local use – are JASSA (http://www.jassa.fr), GPS-SUMO 2.0 (https://sumo.biocuckoo.cn) and DeepSUMO (https://deepsumo.renlab.org). JASSA, developed in 2010–2015, is the most classical among the three tools, relying on a Position Frequency Matrix built from aligned experimental motif sequences [69]. Both GPS-SUMO 2.0 [70] and DeepSUMO [71] – published in 2024 – appear to be based on an older tool: GPS-SUMO 1.0 from 2014 [72]. They incorporate modern machine learning approaches, consider features such as accessibility in addition to sequence patterns, and have been trained on a larger curated sets of reported motifs. Each of the three tools, in addition to SIM detection, is able to detect potential SUMOylation sites – a task for which numerous further tools exist but are not directly relevant to this review. Beyond the powerful SUMO-specific tools, SIMs can also be predicted with some success with more general web servers dedicated to the identification of short linear motifs such as the ELM resource [73].

Beyond canonical bioinformatics, we predict that new machine learning-based structural prediction techniques, such as AlphaFold [74–76], will play an increasingly important role in the field. At the very least, AlphaFold models can suggest whether an apparent SIM motif detected at the sequence level is likely to be available for binding rather than buried inside a folded protein domain. The new prediction algorithms can also be used to model SUMO:SIM complexes. Of note, AlphaFold has shown promising results not only with interactions between folded domains, but also those involving linear motifs [77–81]. However, when using these tools, close attention must be paid to confidence metrics [82,83], and the results should be validated with experimental methods, ultimately involving experimental structural biology.

Lastly, a valuable contribution to the study of SUMO:SIM interactions has also been made by molecular dynamic simulations, including insights into the stability of anti-parallel vs parallel SUMO:SIM interactions [56,84–87].

## Non-covalent SUMO interactions mediated by the SIM motif and their regulation

**Figure 1:**
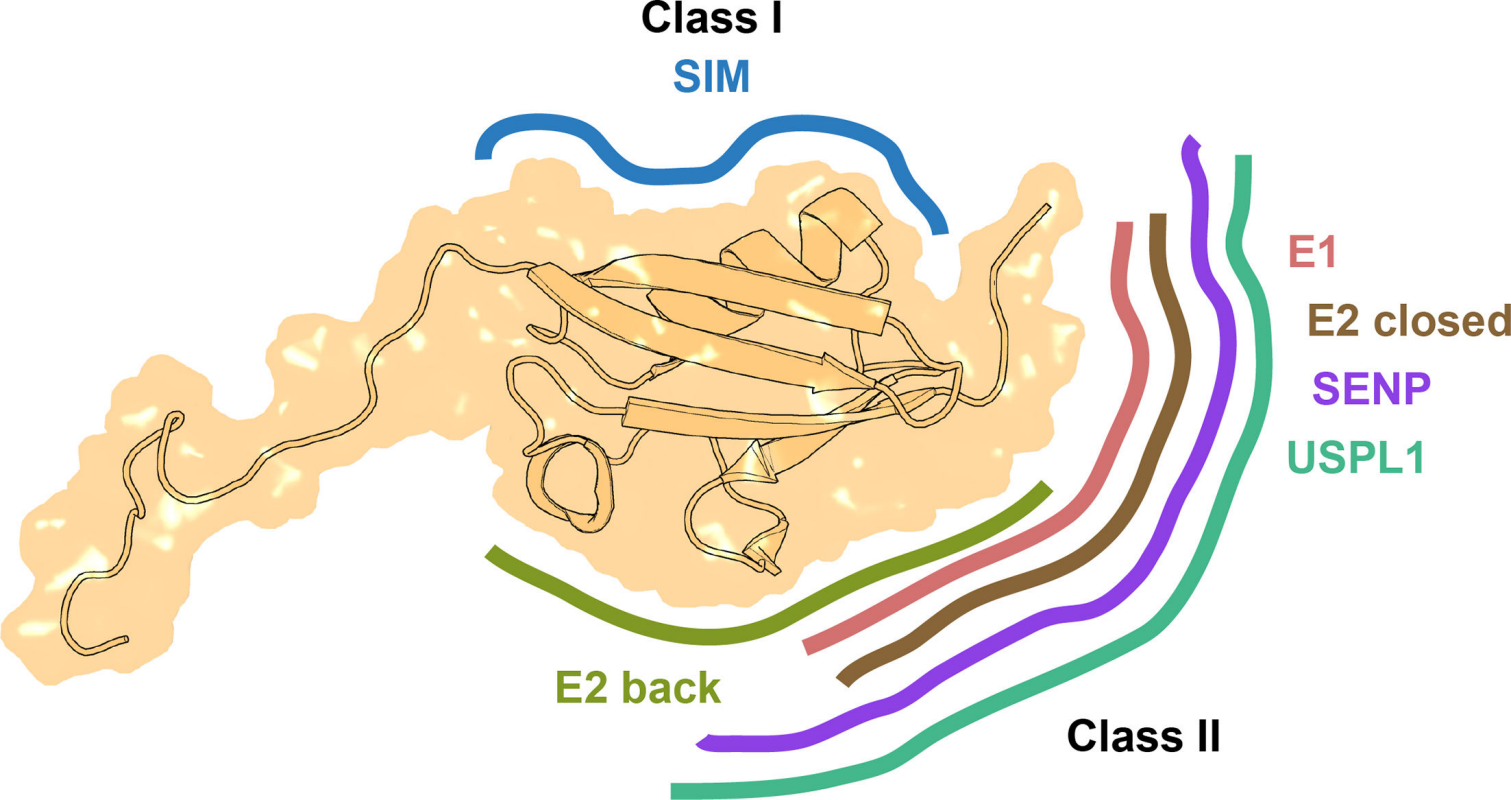
Surfaces on SUMO mediating interactions with SIMs and enzymes. A molecular structure of human SUMO1 (residues 2–97 from PDB 1 A5R, light orange) in transparent space-filling surface representation with surfaces mediating interactions with SIMs and various enzymes schematically indicated. The following interactions are represented: SUMO bound to SAE2 subunit of the E1 enzyme (‘E1’, pink); donor SUMO that is part of SUMO∼UBC9 thioester in the closed conformation, interacting with UBC9 (‘E2 closed’, orange); SUMO non-covalently bound at the back of UBC9 (‘E2 back’, green); SUMO bound to SUMO-specific protease SENP2 (‘SENP’, violet); and SUMO bound by a SUMO-interacting motif (SIM) from RANBP2 (‘SIM’, blue). For more details, see the accompanying review on SUMO:enzyme interactions. The figure is inspired by Figure 2 in the review by Pichler and colleagues [2]

While the non-covalent interactions between SUMO and its cognate E1, E2 and protease enzymes (see the accompanying review [4]) represents canonical protein:protein interactions formed by two partners that are each folded in isolation and associate through complementary surfaces, SUMO can also engage in a different interaction type: with specific short linear motifs typically embedded in disordered regions of proteins and known as SIMs (previously also called SUMO-binding motifs or SBMs) (reviewed in [5–7]). Mediated by a hydrophobic groove at the edge of SUMO’s β-sheet, SIM-mediated interactions, also called class-I SUMO interactions [2], are generally compatible with simultaneous binding to enzymes described in the accompanying review, which involve the opposite side of SUMO (Figure 1).

### History of SIM discovery and SIM definition

A sequence pattern associated with an ability to bind SUMO in a non-covalent manner was first proposed in 2000 following a yeast two-hybrid screen that uncovered interactors of SUMOylated P73 [41]. These proteins shared a conserved Ser-X-Ser sequence (where X is any amino acid), flanked by a hydrophobic region and acidic residues. However, a later study challenged the role of the Ser residues, demonstrating that a cluster of hydrophobic residues was the key determinant for SUMO recognition [57]. This motif was found in several known SUMO pathway components, including SUMO E3 ligases from the PIAS family and RANBP2, and the E1 subunit SAE2. A similar motif was soon identified in yeast proteins as well, underscoring its evolutionary conservation [88].

**Figure 2:**
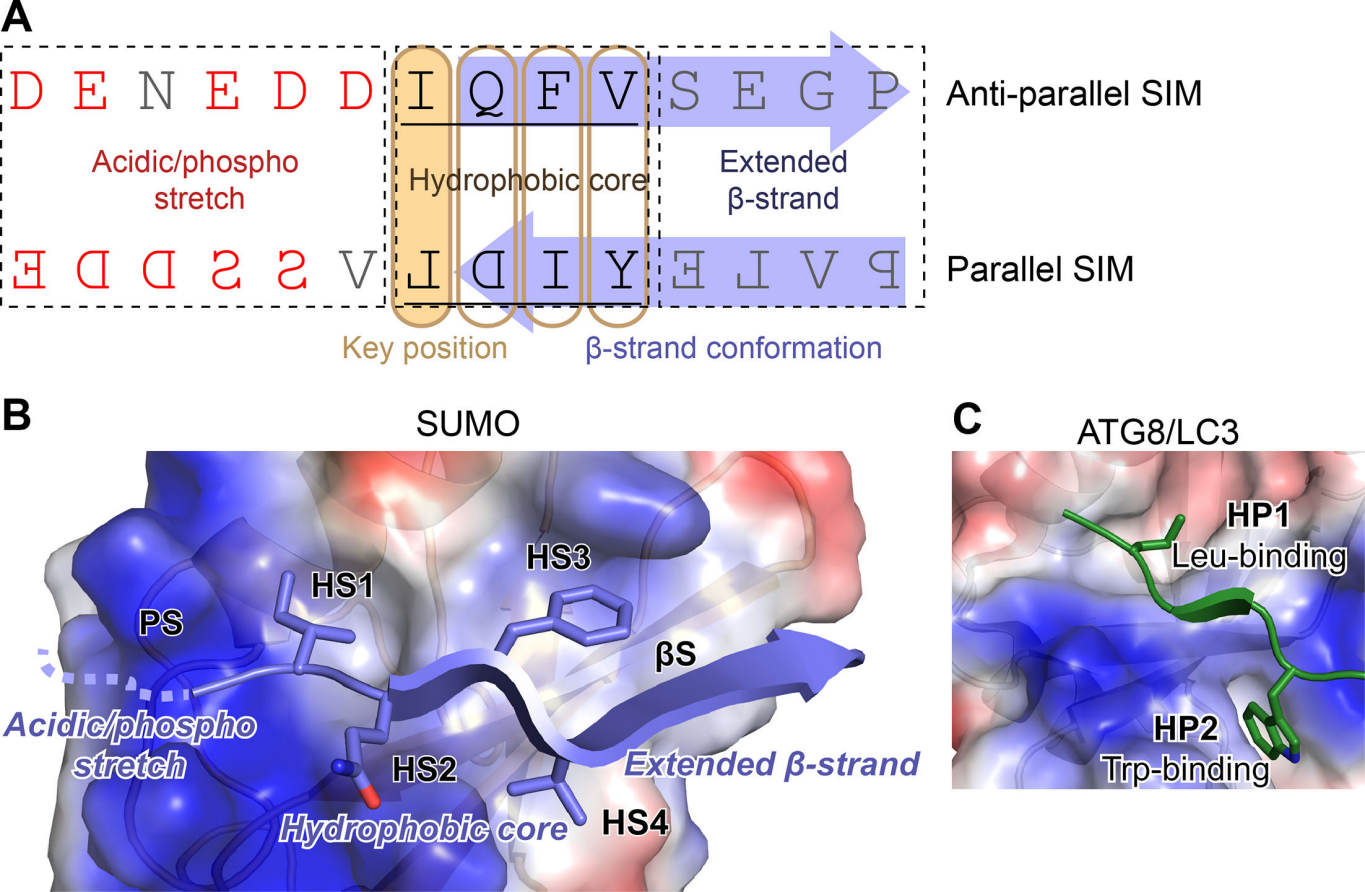
SUMO-interaction motif (SIM) and SUMO:SIM interaction. **A.** Sequences of representative anti-parallel and parallel SIMs (both from human ZNF451, defined based on structural information in PDB entry 5D2M) with labelled functional parts. The sequence of the parallel SIM is written in a mirror-image manner to show that it corresponds to the same pattern except for a reversed orientation. Key hydrophobic positions of the hydrophobic core are highlighted in bold, while acidic (negatively-charged) and phosphorylatable residues are coloured red. The part of the SIM approximately corresponding to a β-strand conformation is indicated with a purple arrow, and the key hydrophobic residue, which docks into the HS1 sub-site on SUMO, is indicated with a light orange rounded rectangle. **B**. SUMO’s SIM-binding groove comprising specific sub-sites. A fragment of a crystal structure showing an anti-parallel SIM from ZNF451 bound to SUMO2 (PDB 5D2M). The SIM is shown as a blue ribbon with the side chains of the amino-acid residues comprising the hydrophobic core shown as sticks. A flexible part of the SIM containing several acidic and phosphorylatable residues – not visible in the crystal structure – is indicated with a dashed line. Distinct SIM parts are labelled in blue italics. SUMO is shown in surface representation coloured according to approximate surface electrostatics calculated using PyMol (blue corresponds to positive charge, red – to negative charge, and white – to uncharged, hydrophobic surfaces). SUMO sub-sites supporting SIM binding are labelled in black bold, using the following acronyms: PS for positively-charged sub-site; HS1 to HS4 for hydrophobic sub-sites 1 to 4, βS for the sub-site accommodating the extended part of the SIM β-strand. **C**. ATG8/LC3:LIR interaction similar to SUMO:SIM interaction. Human LC3 bound by a LIR motif from P62 (PDB 2ZJD) is shown in a similar representation to that in panel B, with LC3 surface coloured according to surface electrostatics. The HP2/Leu-binding site of ATG8/LC3 proteins overlaps with HS1 of SUMO, whereas HP1/Trp-binding site is distinct.

Today, it is well-established that the four-residue hydrophobic core (typically with at least three amino-acid residues with a hydrophobic character, especially Val, Leu or Ile) is the essential element of the SIM, although both acidic (Glu and Asp) and phosphorylatable Ser or, possibly, Thr residues can contribute to binding, as can a region of the protein on the other side of the hydrophobic core, which extends the β-strand formed by the core (Figure 2A). Anti-parallel and parallel SIMs conform to a similar overall pattern except that the order of the elements is reversed in the parallel SIM, consistent with it binding to the same pocket on SUMO but in a reversed orientation. The SIM can be described as a ‘loose’ motif, insofar as the identified particular SIMs do not strictly adhere to one consensus. As mentioned in more detail below, SIMs can bind SUMO in a partly paralogue-specific manner [42].

### Thermodynamic and kinetic properties of SUMO:SIM interactions

The equilibrium dissociation constants, *Kd*s, of canonical SIMs remain moderate, typically in the low to medium micromolar range [57,59,61,89,90] but are generally lower (i.e., the binding they describe is stronger) than those of ubiquitin-binding motifs and domains, which tend to be in the high micromolar range [91]. The kinetic aspects of the SUMO:SIM interactions remain poorly explored, with only one relevant study available to our knowledge to date, which relied on relaxation NMR to estimate the association and dissociation rate constants to be around 2.5 × 10⁵ M⁻¹·s⁻¹ and 8 s⁻¹, respectively, for one particular SIM [65]. These rates indicate that the SUMO:SIM interactions display rapid, reversible kinetics consistent with dynamic protein assemblies.

### Structural basis of the SUMO:SIM interaction

Numerous structural studies have shown that SIMs typically bind to the SIM-binding groove of SUMO through β-strand addition (β-sheet augmentation) [92] (Figure 2B), in which a part of the SIM extends SUMO’s β-sheet by forming an additional strand aligned in either a parallel or anti-parallel orientation [58,59,66,89,93,94]. In an anti-parallel SIM, the β-strand conformation usually starts from the second residue of the hydrophobic SIM core and extends for up to six or so residues; in a parallel SIM, the β-strand conformation tends to start before the hydrophobic core and end at the third residue of the hydrophobic core (Figure 2A). Most SIMs are likely to bind preferentially or even exclusively in one orientation, but in at least two cases a SIM was observed to dynamically switch between both orientations [35,95]. Notably, while the SIM motif is often disordered in its unbound state [96] and undergoes a folding-upon-binding transition, SUMO itself remains largely conformationally stable, with minor adjustments upon SIM binding, primarily in side-chain positions.

A hallmark of interactions that occur through β-strand addition is the formation of backbone hydrogen bonds. Although HDX (hydrogen–deuterium exchange) experiments indicate that at least some SIM peptides mixed with SUMO lack slowly exchanging backbone amide protons often observed in β-sheet structures [57], this may reflect the dynamic and reversible nature of the SUMO:SIM interaction, rather than the absence of backbone hydrogen bonds in the bound state. By involving the main chain of the bound motif, β-strand addition is largely sequence-independent (aside from the fact that some amino acids favour and others discourage a β-strand conformation) [92], which may explain the relative tolerance for sequence variation within SIMs, especially downstream of the hydrophobic core. Perhaps owing to the larger number of hydrogen bonds theoretically formed in an anti-parallel compared with a parallel β-sheet, anti-parallel SIMs appear to form more stable interactions with SUMO, accommodate a broader range of possible sequences, and could be more prevalent in nature [64,84].

### The SIM-binding groove and its sub-sites

**Figure 3:**
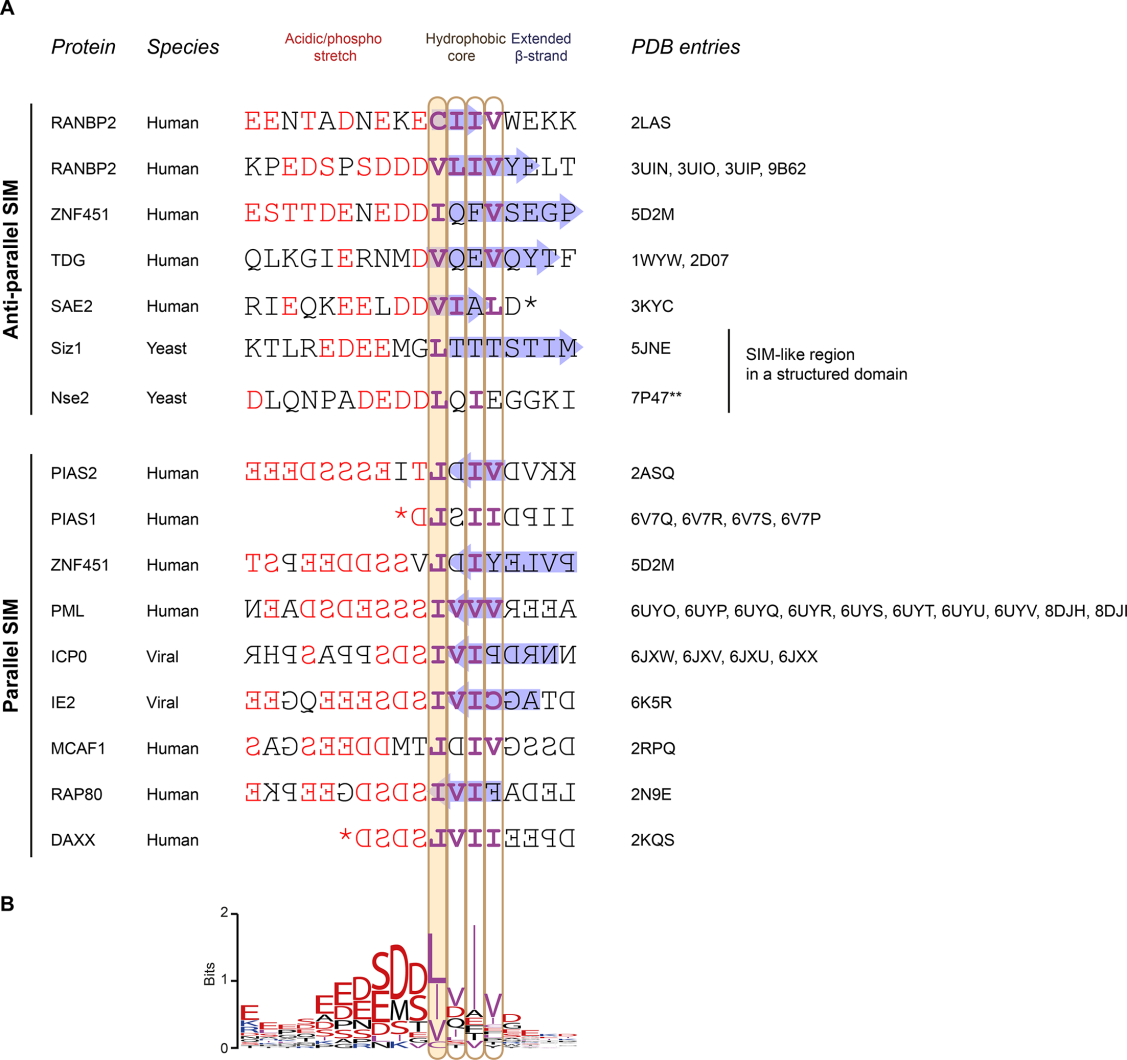
Sequences of structurally validated SIMs. **A.** Sequences of natural linear SIMs which were experimentally visualised in a SUMO-bound state using NMR, crystallography, or cryo-EM. SIMs from RNF4 (PDB 2MP2) and the second SIM of Nse2 (PDB 7P47) were excluded due to the models’ limited accuracy. SIMs are grouped by orientation (anti-parallel above, parallel below). Acidic and phosphorylatable residues are coloured red only in the parts of SIMs corresponding to the acidic/phospho stretch, while the preferred hydrophobic residues (Val, Leu, Ile, and Cys) are coloured purple only when featuring in the hydrophobic core part. SIM residues adopting a β-strand conformation are highlighted with a blue arrow. SIMs lacking any β-strand conformation likely reflect inaccuracies in the geometry of the corresponding structural models rather than the real absence of this conformation. An asterisk (*) marks the C terminus, whereas a double asterisk (**) next to PDB 7P47 indicates that the register of the SIM-like region in Nse2 was shifted by us by one residue relative to the PDB model, following a high-confidence AlphaFold prediction. **B**. A sequence logo created from the sequences aligned in panel A using weblogo 2.8.2 [97]

Nonetheless, amino-acid side chains located at key positions remain essential to the SUMO:SIM interaction, by helping to avoid steric clashes and enabling favourable contacts with several partially distinct sub-sites on SUMO. These contacts include desolvation-driven, van der Waals interactions mediated by hydrophobic residues, along with ionic and polar interactions involving acidic or phosphorylated residues. Based on available NMR and crystal structures of SUMO:SIM interactions, we propose to define five key sub-sites within the SIM-binding groove. These comprise four hydrophobic sub-sites (HS1–HS4), each engaging one residue of the hydrophobic SIM core, a positively charged sub-site (PS) for binding acidic and/or phosphorylated SIM residues, and a β-strand sub-site (βS), where the β-strand can extend in some SIMs, but without strong preference for specific sequence (Figure 2B). Sequences of structurally validated natural linear SIM are presented in Figure 3A, supporting some of the generalisations provided below.

In the order corresponding to that of the bound anti-parallel SIM residues (Figure 2A), the SIM-binding groove begins with the loosely defined PS, which can accommodate several acidic or phosphorylated residues that typically precede the hydrophobic SIM core in anti-parallel SIMs or follow the core in parallel SIMs. The key SUMO residues here are Lys39 and Lys46, which are conserved across paralogues, acting like ‘prongs’ on either side of the groove to co-ordinate acidic or phosphorylated residues through electrostatic interactions. Additional residues might contribute to PS in a paralogue-specific manner, including His43, Lys45, Asn74, Lys78 in SUMO1, and Arg36 and His37 in SUMO2/3 (corresponding to positions 40 and 41 in SUMO1), perhaps explaining differential paralogue preference of some SIMs containing acidic/phosphorylatable stretches [42]. The acidic SIM residues expected to interact with this site often remain unresolved in crystal structures, suggesting they remain flexible or adopt multiple conformations when bound; this seems to be less the case for the phosphorylated residues [59,94].

PS is followed by hydrophobic sub-sites HS1–HS4 (Figure 2B), which begin with HS1, a pocket formed mainly by the following SUMO side chains: Leu47, Val38, the aliphatic part of Lys46, and – to a smaller extent – Thr42 and Phe36. HS1 is arguably the deepest of the three hydrophobic sub-sites and may be the primary from a functional perspective. Constitent with this notion, the SIM residues that dock in HS1 (that is, the first residue of an anti-parallel SIM or the fourth residue of a parallel SIM core) are invariably or almost invariably hydrophobic – especially Leu, Ile or Val – whereas the other hydrophobic-core positions seem more permissive (Figure 3A and B). In at least one case, the SIM residue docking in HS1 is a Cys, but this is consistent with the underappreciated hydrophobic nature of this amino acid [98]. HS1 might be the most evolutionarily ancient of the SIM-binding sub-sites, as suggested by its presence in certain other UBL modifiers, namely those from the ATG8/LC3 family, where it is known as the hydrophobic pocket 2 (HP2) or the Leu-binding site and is critical for recognising LIR motifs [99,100] (Figure 2C). The other ATG8/LC3 site essential for LIR binding – hydrophobic pocket 1 (HP1), also known as the Trp-binding site – partly overlaps with SUMO’s HS4, but is deeper and specialised for recognising large aromatic side chains.

SUMO’s HS1 is followed by three hydrophobic sub-sites that bind the remaining SIM core residues. HS2 lies at the opposite side of the groove from HS1 and forms a shallow pocket between the aromatic ring of Tyr21 and the aliphatic chain of Lys37. HS2 can sometimes accept polar residues, which underlies the common definition of SIMs as ψ-X-ψ-ψ in anti-parallel or ψ-ψ-X-ψ in parallel orientation, where ψ is hydrophobic and X (at HS2) is any residue. Yet, hydrophobic residues are still often preferred at this position (Figure 3B). HS3 is located at the same side as HS1 and is primarily defined by Phe36, alongside Ile34, Arg54 and Ser50. It appears to be the second most selective sub-pocket for hydrophobic residues after HS1 (Figure 3B), perhaps particularly when SIMs bind in a parallel orientation (Figure 3A). HS4 is again situated at the opposite side of the groove and is delimited by the aliphatic portions of the Lys23, Lys37 and His35 side chains.

Lastly, we use the abbreviation βS for the part of the SIM-binding groove with an exposed β-sheet where the SIM motif can interact further through β-strand addition, without specific consensus sequence requirements (Figure 3A and B).

### Regulation of SIM binding by SIM phosphorylation and SUMO acetylation

A well-attested mechanism of SIM regulation is the phosphorylation of Ser (or perhaps sometimes Thr) residues that flank the hydrophobic core in some cases, leading to the augmentation of affinity, typically by at least an order of magnitude [90,101–103]. The molecular basis for this gain probably lies in the greater capacity of phosphoryl groups to form interactions with residues of the PS site defined above relative to unphosphorylated Ser residues or even Asp or Glu residues.

The commonly invoked – and arguably the most intuitive – paradigm for understanding SIM phosphorylation frames it as a switch-like mechanism controlling SUMO binding in response to stress or other signalling cues [90,101]. Indeed, the comparison of unmodified and phosphorylated peptides *in vitro*, or of WT and Ser-to-Ala mutated SIM proteins in cells, suggests that phosphorylation may in some cases effectively ‘license’ barely functional SIMs for SUMO binding, with SUMO serving as a phosphorylation ‘reader domain’ mediating phosphorylation-dependent, inducible interactions [90,101]. This paradigm might be applicable to some cases but should be treated with caution. As the phosphorylatable residues within SIMs are often surrounded by acidic residues, they are favourable targets for the CK2 kinase, which has been shown to phosphorylate certain SIMs [90,101,104] and may be responsible for modifying many others. Given that CK2 is an unusually active kinase capable of constitutively maintaining very high levels of phosphorylation (even approaching 100%) of at least some of its substrates [105], SIM phosphorylation may represent a stable feature of certain SIMs rather than a switch triggered by specific conditions.

Of interest, phosphorylation has also been linked to SUMOylation in other ways: it contributes to enhanced SUMOylation of some SUMOylation sites by promoting their interaction with UBC9 (in this case, the phosphorylation is catalysed by signalling- or cell cycle-dependent Pro-directed kinases) [106,107]; and it regulates SUMO’s half-life and possibly other properties through SUMO phosphorylation [16,28]. See also the next section for the discussion of the potential effects of N-terminal SUMO phosphorylation on SIM binding.

Lastly, the SUMO:SIM interaction can also be modulated by another PTM, acetylation, this time affecting the positively-charged SUMO residues that contribute to the PS site [29,108]. Acetylation on these sites differentially affects binding of various SIMs, notably including phosphorylated ones.

### The effect of SUMO1’s N-terminal tail and zinc on SIM binding

Although SIMs bind the folded core of SUMO, recent studies show that the flexible N-terminal tail of SUMO1 can negatively influence this interaction [56,61,109]. Deletion of the first sixteen residues considerably enhances binding of some SIMs, identifying the SUMO1 tail as an inhibitory element [56,61]. This mechanism may affect different SUMO paralogues and SIMs unequally and appears particularly relevant for phosphorylated SIMs [61]. Two acidic residues in the SUMO1’s tail, Glu11 and Asp12, appear key for this process, occluding the SIM-binding groove by dynamically interacting with surrounding basic residues. Molecular dynamics and phosphomimetic mutagenesis suggest that phosphorylation within the N-terminal tail may amplify this inhibitory effect in SUMO1 and potentially induce it in SUMO2/3, where it is otherwise absent.

A further twist emerged from a serendipitous discovery that, in the presence of zinc ions during crystallisation, the N-terminal tail of SUMO1 bound to a phosphomimetically-mutated SIM from PML partly adopts a helical conformation and undergoes a zinc-mediated interaction with SIM’s phosphomimetic residues [61]. The zinc ion appears to be co-ordinated by Glu11 and Asp15 on SUMO1. Consistently, zinc ions also seem to relieve the inhibitory effect of the N-terminal tail on SIM binding in solution. In this structure, another zinc ion mediates a further interaction between the folded part of SUMO1 and an acidic residue on the other side of the hydrophobic SIM core. It is not known whether zinc is a physiologically relevant general regulator of SIM binding *in vivo*.

### SIMs as fast-evolving short linear motifs

SIMs are a prime example of short linear motifs (SLiMs) – simple protein motifs, typically fewer than ten amino acids in length, that mediate a substantial proportion of interactions within eukaryotic protein networks [110]. SLiMs exhibit remarkable evolutionary plasticity: because their function often depends on just a few key residues, they can arise *de novo* (*ex nihilo*) through only one or a few point mutations – emerging independently in different protein lineages – and be lost just as easily [111]. Hydrophobic SLiMs, such as SIMs, may arise particularly readily owing to a possible bias favouring hydrophobic substitutions during spontaneous mutagenesis [112].

The recurrent independent emergence of similar SLiM patterns in different partners is driven by the nature of the target protein – in this case, SUMO – possessing a binding site with a preference for certain matching sequences. When the target protein is both abundant and biologically important, as SUMO is, and its binding site particularly ‘sticky’ – that is, well adapted to form energetically meaningful contacts even with imperfectly matching sequence elements – such motifs are likely to emerge with particular ease. This may be especially true for binding surfaces containing an exposed β-sheet, as in the case of SUMO’s SIM-binding groove, which allows for β-strand addition with a significant contribution from sequence-independent backbone interactions [92]. Once such an interaction emerges – even if initially weak – it can be refined through selection if it offers a functional advantage. Notably, a protein like SUMO, for which binding motifs can emerge readily, represents both an evolutionary opportunity – by enabling rapid evolution of interaction networks and signalling pathways – and a vulnerability. Indeed, binding motifs may also arise in pathogens such as viruses or parasites, enabling them to interfere with host functions, as seen with pathogen-encoded SIMs discussed later.

As mentioned already, interactions similar to SUMO:SIM can also be formed by some other members of the UBL modifier family, notably by those from the ATG8/LC3- family, which also bind their cognate LIR motifs partly through β-strand addition [99]. It is possible that a rudimentary groove suitable for such binding – while absent from ubiquitin – was present in a common ancestor of some UBL modifiers including SUMO and ATG8/LC3-family proteins.

Another important factor in SLiM evolution is the nature of intrinsically disordered regions, which – being free from the structural constraints of folded domains – can tolerate substitutions more easily and thus evolve more rapidly. Indeed, most SIMs appear to have emerged in such disordered regions. However, binding motifs can also arise within structured scaffolds. To distinguish between the two types, we refer to SIMs embedded in structured domains as ‘SIM-like regions’. In some cases, such motifs may even form from residues that are spatially proximal but not contiguous in sequence, as seen in the one known example of a ‘split SIM-like region’ [113] discussed further below.

## SIM variants: repeated SIMs, extended SIMs, SIM-like regions and split SIM-like regions

The evolutionary creativity of protein systems results in no two SIMs being fully identical. Furthermore, in addition to standard SIMs, different variations on the theme have emerged.

### Repeated SIMs

SIMs are often present in multiple copies within the same protein, with diverse spacing; their function appears to depend on both this spacing and the presence of additional motifs and domains. Frequently, one SIM emerges as dominant, with others playing supportive roles.

The most intuitive role for repeated SIMs is to preferentially bind SUMOylated proteins carrying multiple SUMO modifications – either polySUMOylation or multiSUMOylation – rather than just a single SUMO. This preference should arise because multiple weak SUMO:SIM interactions between a multi- or polySUMOylated protein and a protein with several SIMs, combine to create a strong overall interaction (a co-operativity or avidity effect). Such enhanced binding would not occur with a monoSUMOylated protein. This mechanism appears to apply to the STUbL RNF4, whose N-terminal region contains four closely spaced SIMs [22,33–35,114,115]. The function of STUbLs is discussed in more detail later. The polySUMO-specificity of RNF4 has motivated bioinformatic search for other proteins with closely clustered SIMs as potential further polySUMO binders [50].

While this mechanism is compelling, it is not always evident why a multi–SIM protein should preferentially bind a single polySUMOylated target rather than several distinct SUMOylated proteins at once – especially when SIMs are widely spaced within a large polypeptide, as is often the case, including in STUbLs other than RNF4 [25,26,90]. Simultaneous binding to multiple SUMOylated targets could promote the formation of higher-order networks, potentially leading to condensates, as proposed for SLX4 [116] and discussed further below.

Lastly, a fascinating function for a region composed of just two closely-spaced SIMs is provided by the SUMO E3 ligase ZNF451 [94,117], which is described in more detail below. In this case, the length and amino-acid composition of the spacer between two SIMs is critically important for the interaction and function.

### Extended SIMs

An unexpected way in which a SIM can be extended has been revealed by recent crystal structures of a parallel SIM from PML bound to SUMO1 [59,61,108]. Crystal structures reveal that this SIM contains an Arg-Tyr motif on the other side of the acidic/phosphorylatable region relative to the hydrophobic core. The Arg-Tyr motif folds back on SUMO, with Arg forming a salt bridge with Glu84. As conserved sequence stretches surrounding putative SIM motifs are often longer than the expected minimal SIM composition, it is likely that various SIMs are extended in diverse ways that remain to be explored on a case-by-case basis.

### SIM-like regions embedded in structured domains

**Figure 4:**
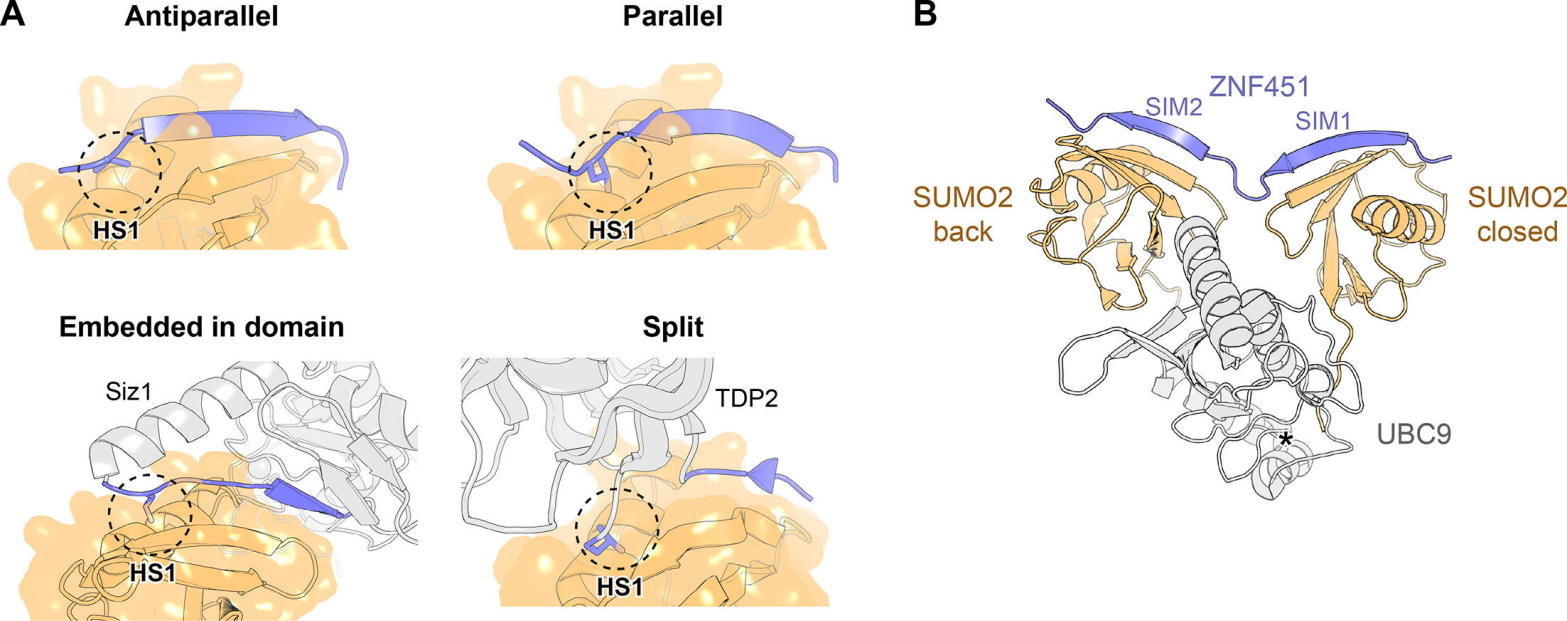
SIM variants and function. **A.** Types of SIM motifs, including an anti-parallel SIM (*upper left*, from ZNF451, PDB 5D2M), a parallel SIM (*upper right*, from ZNF451, PDB 5D2M), a linear SIM-like region embedded in a structured domain (*lower left,* from Siz1, coloured grey with SIM-like region in blue, PDB 5JNE), and a split SIM-like region embedded in a structured domain (*lower right*, from TDP2, coloured grey with SIM-like region in blue, PDB 5TVQ). The key hydrophobic residue in position one or four, respectively, of the hydrophobic core of anti-parallel and parallel SIM is shown as sticks, docked into the HS1 sub-site on SUMO, indicated with a dashed circle. **B**. The closed conformation of the SUMO∼UBC9 thioester stabilised by simultaneous binding of an N-terminal fragment of the human SUMO E3 ligase ZNF451 through two SIM regions to two SUMO molecules, a non-covalent SUMO docked at the back of UBC9 (‘SUMO2 back’) and the donor SUMO that is part of the thioester and which adopts the closed conformation (‘SUMO2 closed’). The structural representation is based on a fragment of PDB entry 5D2M. Cys93 of UBC9 to which the closed-conformation SUMO2 would normally be attached through a thioester bond is indicated with an asterisk.

Going even further beyond extended linear motifs, SIM-like regions can also be embedded within structured domains, with other parts of these domains contributing to SUMO binding. The best-characterised case of this kind is the SP-RING domain, a highly diverged SUMO-specific variant of the RING domain found in SUMO E3 ligases from the Siz/PIAS and related families (hence the name). The SP-RING domain of the yeast E3 ligase Siz1 contains an anti-parallel SIM-like region with a presumably suboptimal Leu-Thr-Thr-Thr hydrophobic core, where Leu docks into the key HS1 sub-site [118] (Figure 4A, *lower left*). However, first, the β-strand that starts with this SIM-like core motif is extended by additional residues, and second, SUMO is also contacted by residues from other structural elements of the SP-RING domain, notably a long α-helix and a zinc-co-ordinating coil region. Together, these contacts amount to a substantial interface of around 800 Å^2^. A distinct, but analogous example is provided by the affimers (adhirons) selected for specific SUMO binding using phage display [119]. While these artificial binders contain SIM-like regions (again, with somewhat imperfect core sequences), their unusually high affinity likely emerges from additional interactions formed by the folded scaffold.

It is conceivable that a further example of a structured domain harbouring a SIM-like region might prove to be provided by certain MYM-type zinc-fingers of proteins ZMYM3 (ZNF261) and ZMYM2 (ZNF198), as the zinc-finger-containing region of these proteins was shown to be both necessary and sufficient for SUMO binding, and the binding appeared dependent on zinc [120]. As these proteins are no longer bound to a SUMO2 mutated at the SIM-binding groove, the binding seems localised to this groove and thus potentially involves a SIM-like region within MYM-type zinc-fingers. However, these speculations should be treated with caution as another study suggested the existence of three canonical SIMs within ZMYM2 – one of them between MYM zinc-fingers – which may account for the binding properties attributed to zinc-fingers themselves [43].

We presume that the prevalence of structured SUMO-interacting domains harbouring SIM-like regions is currently underestimated within eukaryotic proteomes, as SIM motifs are usually searched for bioinformatically in disordered regions and, furthermore, those embedded in structured domains may diverge more strongly from consensus patterns due to compensation by other binding regions.

### Split SIM-like region embedded in a structured domain

A folded scaffold offers a further advantage over a linear motif by theoretically allowing a SIM-like region to form from non-consecutive sequence elements brought together in space. This intriguing mechanism has been confirmed in one known case: that of TDP2, an enzyme that removes topoisomerase II–DNA cross-links and is partly recruited to its targets in a SUMO-dependent manner [113] (Figure 4A, *lower right*). On the opposite side of the catalytic domain relative to the active site, regions immediately upstream and downstream of this domain combine to engage the SIM-binding groove of SUMO2. Here, the specific residues corresponding to the hydrophobic core of a canonical SIM, including Leu370, which docks in the key HS1 sub-site, and Ser121, which protrudes towards HS2, are widely separated in sequence. With additional contacts contributed by the catalytic domain itself, the interaction surface reaches around 800 Å², larger than in typical SUMO:SIM interactions, supporting a higher than usual, submicromolar affinity. Again, further similar cases might exist in nature, and they would be impossible to predict through a bioinformatic search for a consensus SIM pattern.

### Is SOB a SIM?

Two human proteins – dipeptidyl peptidase 9 (DPP9) and the STUbL RNF111 (also known as ARKADIA) – contain similar motifs (ESEVEVIHV and DSEVEIVTV, respectively), jointly termed SOB (‘SUMO1-binding’) motifs, which appear to bind specifically to SUMO1 [36,121]. Whether these motifs are fully functionally equivalent remains unclear, but their similarity is striking. The DPP9:SUMO1 interaction was characterised primarily using pulldown and ELISA assays and was shown apparently not to depend on the SIM-binding groove but instead on His75 and Glu67 – residues located at the opposite face of SUMO1 [121]. As a result, DPP9 is cited as a class-II SUMO interactor, distinct from canonical SIM-based, class-I binders. Studying RNF111 was more complex due to the presence of two additional SIMs; mutation of the SOB motif thus could only have a partial effect on binding to, and ubiquitylating, an oligoSUMO fusion protein mimicking a SUMO chain [36]. Nevertheless, this effect was contingent on the presence of SUMO1 in the oligoSUMO fusion, suggesting that RNF111 may specifically recognise SUMO1 present within SUMO chains via the SOB motif. In this case, mutating Glu67 on SUMO1 did not abolish the observed effect, but mutating His75 did.

While both studies include elegant experiments, due to the limitations of the techniques used, we believe that further analysis – ultimately including structural visualisation of these motifs with SUMO1 – will be needed to determine whether these motifs do bind SUMO1 directly and, if so, whether they do so independently of the SIM groove. Notably, both motifs conform well to anti-parallel SIM consensus patterns, suggesting they may represent a SIM subtype rather than a distinct class after all.

## SIM-dependent molecular functions

Ultimately, the optimisation and retention of SIMs in the course of evolution depends on their positive adaptive role, which in turn is related to a variety of functions that they can perform in the cell. While our focus here is on molecular mechanisms rather than higher-level biological functions, it is interesting to recall an early study combining unbiased mutagenesis of either human SUMO1 or SUMO2 with monitoring transcriptional repression – one of the functions of SUMO – which identified a SUMO surface perfectly overlapping with the SIM-binding groove as essential for this process [122].

### Effector binding

The conceptually simplest role for a SIM is to act as a ‘reader motif’ that promotes an interaction between SUMO and downstream effector proteins. Since SUMOylation is known to lead to diverse effects, an important question is how a given SUMOylated protein recruits an appropriate downstream effector. Part of the answer might be that SUMO often strengthens a pre-existing weak interaction. For example, in the case of the yeast protease Wss1, which contains two SIMs, its recruitment to proteins covalently cross-linked to DNA is enhanced by their SUMOylation, but Wss1 is apparently weakly recruited to its targets even in a SUMO-independent manner, as judged by a partial phenotypic rescue by a mutant Wss1 in which SUMO binding is completely abolished [123].

Another specificity-ensuring mechanism might rely on the diversity of possible SUMO signals. A key example where this paradigm seems applicable is the SUMO-targeted ubiquitin E3 ligases (STUbLs) RNF4, which promotes polyubiquitylation and ultimately either P97 (VCP)-mediated disassembly or proteasomal degradation specifically of polySUMOylated substrates, which it recognises through four adjacent SIMs [22,114,115,124,125]. While RNF4 might prefer uniform SUMO2/3 chains, another STUbL, RNF111 (ARKADIA), by possessing a seemingly paralogue-specific SUMO1-binding (SOB) motif (see a previous section about whether this might be a subtype of SIM) in addition to two more conventional SIMs, may achieve specificity for SUMO1-containing (possibly SUMO1-capped) SUMO2/3 chains [36]. A variant of this paradigm is represented by RAP80, a key DNA repair-associated scaffolding protein that integrates SUMO and ubiquitin signalling [18]. RAP80 contains a SIM and tandem α-helical ubiquitin-interacting motifs (UIMs) that together appear to preferentially bind, *in vitro*, to SUMO–ubiquitin–ubiquitin chain fragment in which SUMO is attached to Lys21 of a ubiquitin unit, which is in turn attached to Lys63 on a further ubiquitin unit [40]. RAP80 is thus expected to get recruited to proteins marked with a specific mixed SUMO-ubiquitin signal.

### SUMO E3 ligases

One of the key protein classes in which SIMs and SIM-like regions are observed is SUMO E3 ligases. These proteins act in part by stabilising the otherwise flexible SUMO∼UBC9 thioester in the so-called closed conformation, from which SUMO is more efficiently transferred onto the substrate, most likely by making the thioester bond accessible and well-aligned for nucleophilic attack by Lys acceptor. To support this conformation, SUMO E3 ligases behave like scaffolds that simultaneously bind to the donor SUMO and to UBC9, stabilising them relative to each other in an appropriate orientation. In all SUMO E3 ligases for which a structural understanding is available, the donor SUMO is engaged *via* a SIM motif or a SIM-like region, whereas UBC9 is bound directly and/or indirectly through a second SIM that engages an auxiliary SUMO molecule docked non-covalently – but rigidly – at the back of UBC9. Through these binding events, the E3 ligase scaffold imposes a favourable geometry for efficient SUMO discharge.

The ligase RANBP2 in its IR1 region sufficient for SUMO E3 ligase activity has only one SIM – for binding the donor SUMO – and it binds UBC9 wholly directly, through a RANBP2 region that is partly helical and partly adopts a coil conformation that wraps around the back of UBC9 [66,126]

The Siz/PIAS and related Nse2/Mms21 families – likely the main SUMO E3 ligases from yeast to humans – contain a SUMO-specific version of the RING domain, known as the SP-RING [11]. The canonical RING domain, stabilised by two zinc atoms, is a signature feature of ubiquitin E3 ligases, where it inserts between ubiquitin and the E2 enzyme to stabilise the ubiquitin∼E2 thioester in its closed conformation [127,128]. SP-RING performs a similar function in SUMOylation by binding both SUMO and UBC9 simultaneously, but it has diverged significantly from the canonical RING domain, co-ordinating only one zinc ion and featuring partly distinct secondary structural elements [129]. Importantly, as already mentioned above, SP-RING contains a SIM-like region that engages donor SUMO through a SUMO:SIM-like interaction [53,118]. The direct binding of the SP-RING domain to UBC9 – unlike that of the UBC9-binding region of RANBP2 – does not occlude the back SUMO-binding site on UBC9, which is thus available for accommodating an auxiliary SUMO molecule. This second SUMO is usually likely bound by a SIM motif located in all SP-RING-containing ligases in a flexible extension downstream of the SP-RING domain, resulting in further stabilisation of the closed conformation [53,118].

Another well-characterised SUMO E3 ligase example demonstrates that just two SIMs – if correctly spaced – can be sufficient for SUMOylation catalysis, without the need for a structured element [94,117]. The N-terminal region of the ligase ZNF451 contains two SIMs that play non-redundant roles in this process, one engaging the donor SUMO and the other binding to the auxiliary SUMO non-covalently docked at the back of UBC9, effectively tethering the donor SUMO and UBC9 together in the closed conformation (Figure 4B). The eight-residue spacer between the two SIM cores plays a critical role in this process by ensuring the correct distance and making an additional direct interaction with Asp19 of UBC9.

In addition to SIMs and SIM-like regions directly involved in the SUMOylation mechanism, SUMO E3 ligases often contain further SIMs within their disordered extensions [60,64], which may serve to maintain attachment to the SUMOylated product and potentially protect it from proteolysis, and/or to mediate localisation to SUMO-rich cellular compartments [130–132].

### SIM-dependent substrate SUMOylation

While SIMs located in SUMO E3 ligases promote the SUMOylation of substrates in *trans* – that is, from within a separate protein (the ligase) – SIMs can also enhance SUMOylation in *cis*, when present within the substrate itself. More precisely, what has been observed is that several SIM-containing substrates – including (but not limited to) human TDG [133], human DAXX [47], human USP25 [134], human BLM [135], and yeast Srs2 [136] – are less efficiently SUMOylated when their SIM is mutated. The mentioned observations have been made *in vitro* and/or in cells, depending on the substrate. Negative examples where SIM deletion has no effect on SUMOylation are scarce (possibly the case for human SP100 [137] but see the later part of this section for details), which could suggest that it is a fairly general mechanism that might apply to most SIM-containing substrates. Whether this is the case and whether it further implies that SIM-containing substrates are on average more highly SUMOylated than SIM-deficient ones is not known, although this question could likely be resolved bioinformatically based on existing SUMOylation datasets.

The observed effect is generally thought to result from the SIM facilitating recruitment of the substrate to the SUMO∼UBC9 thioester [47,134–136], with the SIM potentially binding either the donor SUMO or the second SUMO molecule non-covalently docked at the back of UBC9. This mechanism could, however, be confounded by the presence of SUMO molecules not associated with UBC9, which would be expected to compete for SIM binding; indeed, in the case of yeast Srs2, binding to SUMOylated proteins has been shown to inhibit SUMOylation [136], suggesting that the situation in cells – where SUMO is linked to multiple distinct factors – is likely more complex than *in vitro*. On the other hand, in at least one case, that of yeast Yen1, the effect could not be recapitulated *in vitro* but was observed in cells, suggesting that SIMs may also enable SUMOylation by governing the subcellular localisation of a substrate rather than simply helping recruit it to UBC9 [138].

Given that certain SIMs show a binding preference for SUMO2/3 over SUMO1 or vice versa [42], the *cis*-acting mechanism might promote SUMOylation in a paralogue-specific manner, as reported for some substrates [134,135]. Consistent with the SUMO-binding properties of SIMs affecting the efficiency of SIM-dependent SUMOylation, SIM-dependent substrates show enhanced SUMOylation with N-terminally truncated SUMO1 (which appears to be generally bound more strongly by SIMs) compared with full-length SUMO1 [139].

Lastly, when UBC9 is pre-SUMOylated on one of its Lys residues (apparently predominantly Lys14 in human UBC9), or artificially fused to SUMO, it becomes particularly efficient at SUMOylating SIM-containing substrates, showing enhanced SIM-dependent modification even of those, such as human SP100, whose SUMOylation by unmodified UBC9 appears SIM-independent [137,140]. This provides another potential layer of complexity, where UBC9 SUMOylation could regulate substrate selection.

### SUMO as a molecular glue and the SUMO spray concept

In a dynamic cellular context, SUMO conjugation is frequently spatially and temporally regulated. For instance, in the context of DNA repair in yeast, local recruitment of SUMO ligases to DNA lesions appears to generate a concerted ‘SUMO spray’, coating multiple nearby proteins with SUMO [130].

This localised surge in SUMOylation is further linked to the concept of SUMO as a ‘molecular glue’ – a mechanism for potentiating protein:protein interactions [130]. When a protein is modified with SUMO, these SUMO moieties can engage SIMs on nearby proteins, thereby accelerating and stabilising the formation of multi-protein complexes and other assemblies at a given location. In this framework, SUMO:SIM contacts may either trigger new interactions or reinforce interactions between proteins that, without SUMO, have only weak intrinsic affinities and/or slow association rates.

The concepts of ‘SUMO spray’ and ‘molecular glue’ imply that the functional impact of SUMOylation might not always be – or perhaps rarely is – attributable to individual sites on single proteins [141]. Rather, analogous to the phenomenon of genetic redundancy – where perturbations often have little effect until many accumulate – SUMOylation may involve modifications at many, partly redundant, sites acting collectively to make the response to specific conditions such as DNA damage or other types of stress more efficient or robust. However, while these concepts are very promising, they still remain little tested beyond the original context of yeast DNA repair [130]. A further example where SUMOylation might have a group effect on multiple proteins is the action of STUbLs such as RNF4, particularly in the downregulation of the SUMOylation machinery itself [142]. Finally, some general support to the notions of spray-like protein group SUMOylation and the molecular glue function is provided by the observations that, at a systems level, SUMOylated proteins tend to form functionally and physically interconnected networks [17,143].

### Condensate formation

A specific case of the ‘SUMO glue’ concept concerns the effect of SUMO:SIM interactions on the formation of large protein networks that can demix from their environment, manifesting as condensates. From a topological point of view, the formation of such networks requires that an individual molecule can simultaneously interact with three or more others [144]. This is theoretically the case for proteins that have both multiple SUMOylation sites and multiple SIMs, allowing them to connect with multiple partners simultaneously through SUMO:SIM interactions. Owing to their low-affinity, dynamically reversible character, SUMO:SIM interactions are expected to confer on such networks potentially beneficial fluidity. An artificial demonstration of SUMO:SIM-dependent condensate formation has been obtained by mixing two designed proteins, one composed of multiple fused SUMOs and the other harbouring multiple SIMs [145]. Natural examples of condensates that depend at least in part on multivalent SUMO:SIM interactions include SLX4-mediated condensates [146] and PML bodies [147], although the role of SUMO:SIM interaction in the formation of the latter is controversial [148]. SUMOylation has also been implicated in the condensation of the transcriptional regulator NELF during stress, but it is not clear whether this effect depends on SUMO:SIM interactions [149]. The ability to nucleate condensate formation by such proteins is often amplified by their SIM-independent oligomerisation mediated by dedicated structured oligomerisation domains acting as ‘valency multipliers’ [150], as seen in both SLX4 [151] and PML [152,153]. SUMO:SIM interactions not only drive condensate formation but also, and perhaps primarily, mediate the recruitment of ‘client’ proteins to a condensate – as seen with the SIM-dependent localisation of proteins such as DAXX and SP100 to PML bodies [90,148,154]. Lastly, SUMOylation can apparently also play an opposite role, by preventing certain proteins from clustering or aggregating together – as expressed in the idea of SUMO as a ‘solvent’, as well as a glue [155].

### SIMs in proteins of viruses and parasites and in anti-viral immunity

To proliferate efficiently, viruses often interfere with the PTM machinery of the host – not only to support replication but also to evade immune defences. Viral interference can range from simply inhibiting PTMs to actively exploiting them for the virus’s benefit. SUMOylation appears to be one of the PTMs frequently targeted by viruses [156,157], with many viral proteins containing putative SIMs. Although none of these viral SIMs has yet been directly visualised in complex with SUMO *via* structural biology, many sequences closely match the SIM consensus motif, and several have been shown to interact with SUMO or be functionally linked to SUMOylation.

The following few examples illustrate the diversity of SIM-dependent functions in viruses. In Dengue and Zika viruses from the *Flaviviridae* family, a putative SIM has been identified in the NS5 protein. In Dengue virus, this SIM is essential for NS5 SUMOylation, which in turn enhances protein stability and positively affects viral replication [158]. In Zika virus, mutation of this SIM impairs NS5’s ability to inhibit type-I interferon signalling.

The ICP0 protein of Herpes simplex virus type 1 (HSV-1), identified as a potential STUbL, contains a SIM whose affinity for SUMO increases upon phosphorylation [103]. ICP0 promotes degradation of SUMOylated host proteins such as PML and SP100. Additionally, Kaposi sarcoma-associated herpesvirus expresses K-bZIP, a putative SUMO2/3-specific E3 ligase targeting the host tumour suppressor p53 to promote viral replication; this activity depends on a SIM motif within K-bZIP [159]. Two SIMs have also been proposed in the LANA protein of the same virus, apparently facilitating LANA’s SUMOylation and its role in viral persistence [51]. Finally, in Epstein-Barr virus, the viral kinase BGLF4 contains two putative SIMs essential for its nuclear localisation and prevention of nuclear export [160]. The pro-viral functions of BGLF4, including dispersal of PML bodies and enhancement of viral replication, depend strictly on the integrity of these SIMs as well as its kinase activity. Collectively, the various SIMs identified in viruses underscore that the SIM-binding groove on SUMO represents both a functional advantage and a vulnerability that can be exploited by viral virulence factors.

Interestingly, SIMs can not only help viruses infect hosts but are also found in host immunity pathways. Recently, researchers found a new human SMC5:SMC6 complex subunit, SIMC1, which contains SIMs and helps recruit the SMC5:SMC6 complex to viral episomes to restrict viral replication, demonstrating a previously underappreciated role of SIMs in anti-viral responses [161].

## SIM-independent effector binding

It is difficult to determine to what extent SIMs and their variants are truly the most abundant SUMO-binding elements (although this is likely the case, given that SLiMs of this kind tend to emerge relatively rapidly in evolution), and to what extent their apparent prevalence reflects the fact that they can be readily predicted bioinformatically, whereas identifying other binding modes would require other approaches. In any case, SIM-independent SUMO-binding modes have also been described – not only including those with core SUMOylation enzymes, but also extending to effector interactions. While recent proteomics studies aimed at identifying such alternative interactions suggest they may also be relatively common [8,9], definitive evidence – including experimental structures of complexes with SUMO – is largely lacking, highlighting the need for further investigation.

### ZZ zinc-finger domains

Two interesting candidates for SIM-independent SUMO-binding effector modules are ZZ zinc finger domains from two distinct human proteins, HERC2 and CBP [162–164]. It is possible (though not yet experimentally tested) that these findings reflect a general broader feature of ZZ domains, present in about 20 human proteins; at the very least, they are likely to extend to P300, which is closely similar to CBP. The ZZ-mediated binding appears to occur in the low micromolar range for SUMO1 (for both the HERC2 and CBP domains) and approximately an order of magnitude lower for SUMO2 (measured only for the HERC2 domain). The interaction appears to be zinc-dependent, indicating it requires correctly folded ZZ domain, although its precise mapping on the ZZ domain remains somewhat inconclusive.

An NMR chemical shift perturbation study aimed at identifying the regions of the CBP ZZ domain and SUMO1 involved in the interaction identified residues 1695–1702 in the CBP ZZ domain (corresponding to 2735–2742 in the HERC2 domain), and Lys16, His43, Lys46, His75, Lys78 and Glu83 within SUMO1 as candidates [162]. However, mutating the pinpointed side chains on CBP did not lead to decreased SUMO1 binding affinity, calling into question their major role in the interaction. In contrast, two SUMO1 mutants encompassing subsets of the identified residues each abolished the interaction, supporting the identification in this case. The implicated SUMO1 residues map to an area largely distinct from the SIM-binding groove, near the region from which the N-terminal tail protrudes.

Recently, an unusual crystallographic approach involving a fusion of the six N-terminal residues (excluding the initiator methionine) of either SUMO1 or histone H3 to the HERC2 ZZ domain was used to recreate – between neighbouring molecules in the crystal lattice – a non-covalent interaction between these N-termini and the same binding pocket on the ZZ domain [164]. The involvement of the same ZZ pocket is striking, as the N-termini of SUMO1 and histone H3 have completely different sequences (SDAEAK vs ARTKQT). A key contact involves Asp residues 2709, 2728, and 2730 of HERC2 (residues corresponding to Asn1708, Asp1725 and Asp1727 in CBP) and the N-terminal residues of SUMO1/H3. While this mode of binding raises questions about sufficient specificity of these interactions to be biologically meaningful, solution experiments from the same study demonstrated that short N-terminal fragments of both SUMO1 and histone H3 are sufficient for micromolar-range binding to the ZZ domain.

Assuming that the ZZ domains of CBP and HERC2 bind SUMO1 in a similar manner, the NMR study involving the CBP ZZ domain and the crystallographic study involving the HERC2 ZZ domain could potentially be reconciled by a model in which SUMO1 interacts with ZZ domains primarily through its extreme N terminus (which may have remained unassigned in the NMR study due to flexibility in the unbound state), with additional contributions from the face of SUMO1 from which the N-terminal tail protrudes. Further investigation is needed to conclusively resolve the nature of SUMO:ZZ domain interactions.

### XRCC4

Two recent studies have identified XRCC4 as a SUMO2 binder with a preference for chains, the interaction potentially involving multiple distinct SUMO2-binding sites on XRCC4 [38,39]. Mapping the interaction regions on XRCC4 using various techniques – including carbene footprinting and NMR – and comparing them with the available XRCC4 crystal structures suggests that these interactions do not involve a canonical SIM, which would typically be unstructured in the absence of SUMO. While the binding site(s) within the XLF domain of XRCC4 – which includes an exposed β-strand – could represent a SIM-like motif embedded within a folded domain, one interaction site appears to reside within the helical coiled-coil region of XRCC4, pointing to a non-canonical helical motif for SUMO binding. This should be more conclusively demonstrated using structural biology techniques capable of directly visualising interactions.

### WD40 domains

Further candidates for SIM-independent SUMO binding have been revealed by a recent proteomics analysis by Vertegaal and colleagues, which compared proteins co-precipitated from cell lysate using wild-type SUMO2 *versus* SUMO2 mutated at the SIM-binding groove [9]. While many of the identified potential binders interacted in a SIM-binding groove-dependent manner, a considerable number appeared to bind equally well to wild-type and mutant SUMO2. Notably, this included a group of proteins harbouring a specific type of β-propeller domains: WD40-repeat domains. Interestingly, a β-propeller domain, although not of the WD40-repeat type, features also in the previously reported potential SUMO interactor DPP9 (see section *Is SOB a SIM?* above). However, in that previous case, the binding motif was apparently localised to a hairpin region protruding from the propeller rather than the propeller itself [121].

Focusing on one of the WD40-repeat β-propeller proteins, SEC13, the authors of the recent study observed that the AlphaFold models of the SEC13:SUMO2 complex did not converge on a single preferred relative orientation of the interacting partners, suggesting an uncertain prediction. Nonetheless, the interaction reproducibly involved the same face of the WD40 domain, corresponding to one of the axial surfaces of the propeller, and mutations in the identified residues abolished binding in pulldown assays. On SUMO2, the interaction appeared to involve the C-terminal region; however, deletion of the last nine residues of SUMO2 (a relatively drastic intervention that removes a part of one of the β-strands integral to the SUMO fold) had only a partial effect on SEC13 binding. Overall, while these findings require further investigation and ideally high-resolution experimental structural characterisation, they open fascinating new perspectives on non-canonical SUMO-binding modes.

## Summary and outlook

In this and the accompanying review [4], we aimed to comprehensively discuss non-covalent SUMO interaction, here focusing specifically on those mediated by the SIM and other emerging SUMO-binding elements. Decades of research have shown that SUMO proteins serve as major interaction hubs, capable of engaging with a wide variety of partners. Interactions with the catalytic domains of E1, E2 and SUMO-specific protease enzymes are generally mediated by the β-sheet face of SUMO, whereas most other interactions – including those with disordered extensions of E1 and protease enzymes, as well as SUMO E3 ligases and numerous effector proteins – involve the SIM-binding groove, a signature feature located at the opposite face of the SUMO molecule (Figure 1). Here, we offered a systematic dissection of this critical binding site, defining distinct sub-sites mediating interactions with SIM residues (Figure 2B).

The SIM-binding groove represents SUMO’s ‘sticky epitope’ – a site for which binding motifs appear to emerge relatively readily during evolution, possibly because the groove contains an exposed edge of a β-sheet that enables external elements to bind through β-strand addition in a partially sequence-independent manner. Also, the deep HS1 pocket well-suited to accommodate an elongated aliphatic residue provides an efficient anchoring point (Figure 2A). Indeed, most structurally validated SIMs contain a Leu or Ile in the corresponding position (Figure 3A and B).

SUMO:SIM interactions, by being sterically compatible with various SUMO:enzyme interactions (Figure 1), can serve to stabilise particular complexes and conformations, and govern subcellular localisation of SUMO (de)conjugating enzymes. This is exemplified in the final step of the SUMOylation cascade, where the E2 enzyme UBC9 interacts with two SUMO molecules. The same two SUMOs are engaged, *via* SIMs, by an E3 ligase, which thus brings the SUMOylation machinery into an active, ‘closed’ conformation ready to transfer SUMO onto the substrate (Figure 4B). SUMOylation is like a dance, with SIM motifs guiding the choreography like the hands of a skilled instructor.

Although SIMs are often considered stereotypical elements that ‘always bind in the same way’, recent research has revealed SIM variations such as extended SIMs, SIM-like regions and a fascinating case of a split SIM-like region [113] (Figure 4A). More such non-canonical examples might remain to be identified and characterised. Moreover, SIM-independent binding modes may be more prevalent than currently appreciated. While it seems unlikely that SUMO harbours another, previously unrecognised, groove for accommodating linear motifs distinct from SIMs, it is clear that structured domains can engage SUMO through diverse surfaces – many of which are likely yet to be identified.

Looking ahead, a major challenge will be the continued optimisation of experimental strategies to identify SUMO-mediated interactions, in close co-ordination with increasingly high-throughput computational structure prediction methods. Beyond simple identification of SUMO binders, we will probably see further expansion of recent efforts to address how different proteins recognise diverse SUMO signals, from specific monoSUMOylated substrates to various types of SUMO and SUMO–ubiquitin chains. Lastly, robust validation and detailed characterisation, including quantitative measurements of binding parameters and high-resolution structural techniques, will remain essential for producing insights that are reliable, mechanistically informative and structurally precise.

**Summary**

- The SUMO-interacting motif (SIM)-binding groove of small ubiquitin-like modifier (SUMO), which can be subdivided into several sub-sites, engages protein motifs known as SUMO-interacting motifs (SIMs), which are found in SUMO E3 ligases and a variety of effector proteins such as DNA-repair proteins, SUMO-targeted ubiquitin ligases and cell-signalling components.
- SIM-dependent molecular functions include direct involvement in the SUMOylation machinery (especially SUMO E3 ligases), promoting SUMOylation of SIM-containing substrates, and promoting protein:protein interactions and condensate formation.
- Beyond canonical linear SIMs, which occur in the anti-parallel and parallel orientations, SIM variants include repeated SIMs, extended SIMs, SIM-like regions embedded in structured domains, and split SIMs.
- Not all SUMO:effector interactions. are SIM-dependent, and alternative SUMO modes are emerging from recent studies, although still awaiting full validation and characterisation.
- Proteomics, live-cell imaging, high-resolution structural methods and structural modelling will enable quantitative and temporal mapping of SUMO:SIM dynamics.

## Open Access

This article has been published open access under our Subscribe to Open programme, made possible through the support of our subscribing institutions, learn more here: https://portlandpress.com/pages/open_access_options_and_prices#conditional

## Abbreviations

AIM: ATG8-interacting motif
ATG8: autophagy-related protein 8
BLI: bio-layer interferometry
BLM: Bloom syndrome helicase
BRCA1: breast cancer type 1 susceptibility protein
CBP: CREB-binding protein
CK2: casein kinase 2
DAXX: death domain-associated protein
DPP9: dipeptidyl peptidase 9
E1: UBL activating enzyme
E2: UBL conjugating enzyme
E3: UBL ligase
ELM: eukaryotic linear motif
EM: electron microscopy
HDX: hydrogen–deuterium exchange
HERC2: HECT and RLD domain containing E3 ubiquitin protein ligase 2
HP1 and HP2: hydrophobic pocket 1 and 2 (on ATG8/LC3 proteins)
HS1–HS4: hydrophobic sub-sites 1–4 (in the SIM-binding groove)
ITC: isothermal titration calorimetry
JASSA: joint analysis of SUMOylation sites and SIMs algorithm
Kd: dissociation constant
LIR: LC3-interacting region
MMS21: methyl methanesulfonate-sensitivity protein 21 (yeast SUMO E3 ligase, Nse2)
MYM: myeloproliferative and mental-retardation motif (zinc finger type)
NELF: negative elongation factor complex
NMR: nuclear magnetic resonance
Nse2: non-structural maintenance of chromosomes element 2
P73: protein with apparent weight of 73 kDa (homologue of P53)
P97: protein with apparent weight of 97 kDa, also called VCP (mammalian Cdc48 orthologue)
P300: protein with apparent weight of 300 kDa (histone acetyltransferase)
PDB: protein data bank
PDSM: phosphorylation-dependent SUMO modification motif
PIAS: protein inhibitor of activated STAT
PIAS: protein inhibitor of activated STAT (SUMO E3 ligase family)
PML: promyelocytic leukemia protein
PS: positively-charged sub-site (in the SIM-binding groove)
PTM: post-translational modification
RANBP2: RAN binding protein 2
RAP80: receptor-associated protein 80
RNF: RING finger protein
SBM: SUMO-binding motif
SENP: sentrin/SUMO-specific protease
SIM: SUMO-interacting motif
SLX4: structure-specific endonuclease subunit 4
SLiM: short linear motif
SMC5/6: structural maintenance of chromosomes 5/6 complex
SP100: speckled protein of 100 kDa
SPR: surface plasmon resonance
SP-RING: Siz/PIAS-type RING domain specific to SUMO
STUbL: SUMO-targeted ubiquitin E3 ligase
SUMO: small ubiquitin-like modifier
Smt3: suppressor of Mif two 3 (yeast SUMO orthologue)
TDG: thymine DNA glycosylase
TDP2: tyrosyl-DNA phosphodiesterase 2
UBC9: ubiquitin-conjugating enzyme 9 (SUMO-specific E2 enzyme)
UBL: ubiquitin-like
UFD1: ubiquitin fusion degradation protein 1
UIM: ubiquitin-interacting motif
VCP: valosin-containing protein (P97, mammalian Cdc48 orthologue)
WD40: tryptophan–aspartic acid 40 repeat
WT: wild type
XRCC4: X-ray repair cross-complementing 4
Y2H: yeast two-hybrid
ZMYM: zinc finger MYM-type protein
ZNF: zinc finger protein
ZZ: zinc finger type coordinating two zinc ions
cryo-EM: cryogenic electron microscopy
βS: β-strand sub-site (in the SIM-binding groove)
